# Supplementary material for: Resolving the full spectrum of human genome variation using Linked-Reads
Source: Genome Res. 2019 Apr;29(4):635–45. doi: 10.1101/gr.234443.118 (PMC6442396; doi:10.1101/gr.234443.118)
Supplement: Supplemental Material [file supp_gr.234443.118_Supplemental_longranger-2.2.zip › longranger-2.2.tar.bz2!/lr2.2-public/lib/stan/cmdstan/stan/doc/api/html/coupled__ode__observer_8hpp_source.html]

Stan: src/stan/math/ode/coupled\_ode\_observer.hpp Source File


|  |  |
| --- | --- |
|  | Stan  2.5.0  probability, sampling & optimization |


- Main Page
- Related Pages
- Namespaces
- Classes
- Files

- File List
- File Members

All Classes Namespaces Files Functions Variables Typedefs Enumerations Enumerator Friends Macros Pages

- src
- stan
- math
- ode

coupled\_ode\_observer.hpp

Go to the documentation of this file.

1 #ifndef STAN\_\_MATH\_\_ODE\_\_COUPLED\_ODE\_OBSERVER\_HPP

2 #define STAN\_\_MATH\_\_ODE\_\_COUPLED\_ODE\_OBSERVER\_HPP

3

4 #include <vector>

5

6 namespace stan {

7

8  namespace math {

9

15  struct coupled\_ode\_observer {

16

17  std::vector<std::vector<double> >& y\_coupled\_;

18  int n\_;

19

26  coupled\_ode\_observer(std::vector<std::vector<double> >& y\_coupled)

27  : y\_coupled\_(y\_coupled), n\_(0) {

28  }

29

36  void operator()(const std::vector<double>& coupled\_state,

37  const double t) {

38  y\_coupled\_[n\_] = coupled\_state;

39  n\_++;

40  }

41

42  };

43

44  }

45

46 }

47

48 #endif

stan::math::coupled\_ode\_observer::y\_coupled\_

std::vector< std::vector< double > > & y\_coupled\_

**Definition:** coupled\_ode\_observer.hpp:17

stan::math::coupled\_ode\_observer::n\_

int n\_

**Definition:** coupled\_ode\_observer.hpp:18

stan::math::coupled\_ode\_observer

Observer for the coupled states.

**Definition:** coupled\_ode\_observer.hpp:15

stan::math::coupled\_ode\_observer::operator()

void operator()(const std::vector< double > &coupled\_state, const double t)

Callback function for Boost's ODE solver to record values.

**Definition:** coupled\_ode\_observer.hpp:36

stan::math::coupled\_ode\_observer::coupled\_ode\_observer

coupled\_ode\_observer(std::vector< std::vector< double > > &y\_coupled)

Construct a coupled ODE observer from the specified coupled vector.

**Definition:** coupled\_ode\_observer.hpp:26


---

[ Stan Home Page ]

*© 2011–2014,
Stan Development Team.*
